# Does undertaking rural placements add to place of origin as a predictor of where health graduates work?

**DOI:** 10.1111/ajr.12864

**Published:** 2022-03-24

**Authors:** Timothy C. Skinner, Libby Semmens, Vincent Versace, Melanie Bish, Isabelle K. Skinner

**Affiliations:** ^1^ 2080 La Trobe University Department of Rural Health La Trobe University Bendigo Victoria Australia; ^2^ 4321 Institute of Psychology University of Copenhagen Copenhagen Denmark; ^3^ Centre for Rural and Remote Health James Cooke University Mount Isa Queensland Australia; ^4^ Deakin Rural Health School of Medicine Deakin University Warrnambool Victoria Australia

**Keywords:** education, rural and remote education, Rural health, student placements, workforce

## Abstract

**Objective:**

To determine the work location (metropolitan, regional, rural and remote) of graduates in nursing, allied health and oral health disciplines who complete their professional training, end‐to‐end training, in a regional or rural area noting the potential inclusion of a metropolitan‐based placement for speciality practice not available in rural or regional Victoria.

**Methods:**

We tracked the place of employment from the Australian Health Practitioners Regulation Agency (AHPRA) of all graduates from a regional/rural tertiary education provider. The student home address at enrolment, locations where they undertook all placements and their current place of work were described using an objective geographical model of access, the Modified Monash Model.

**Results:**

Seventy‐five per cent of 5506 graduates were located in the AHPRA database. About one third of graduates were working in metropolitan areas, 1/3 in regional cities and 1/3 in rural areas. Students' origin accounted for 1/3 of variance in current workplace location. The more placement days students completed in regional/ rural areas was also a significant predictor of working in a regional or rural area.

**Conclusion:**

End‐to‐end training in regional/rural areas is an effective approach to retaining a regional/rural workforce. Student origin is a strong predictor of working rural or regionally, as is undertaking placements in rural areas. This suggests that priority for rural/ regional student placements should be given to students in end‐to‐end regional/ rural programs and students from a regional/ rural background.

**What is already known on the subject:**

- Recruitment and retention of health care professionals into rural and regional Australia is a persistent challenge
- Health professionals' decision to work in rural settings is influenced by having a rural background and family connections

**What this study adds:**

- Establishes that the more time individuals spend on placement in a metropolitan area, the less likely they are to work in regional /rural areas
- Identifies that the locations where students did their placement is a significant predictor of their primary place of practice

## 1 INTRODUCTION

Of the Australian population, 20% lives permanently in rural areas (Modified Monash Model 3–7).^1^, ^2^ The trend in population mobility has seen regional Australia attract more people than it lost to capital cities.^3^ Consecutive census data from the previous decade affirm more than 1.2 million people either moved to regional Australia or moved around regional Australia from one location to another.^3^ Regional, rural and remote communities include primary producers, Indigenous Australians and others who contribute to the rich fabric of the nation. These individuals and communities need access to health, social and education services the same as the rest of the Australian populations.

The allocation of state government funding has responded to this with a clear focus on building infrastructure in rural and regional Victoria to enable access to localised, timely health care. One of the main challenges to providing these services is the recruitment and retention of health care professional staff to these services. In response, the Australian Government has established a long‐term funding scheme, currently referred to as the Rural Health Multidisciplinary Training (RHMT) program. One of the main goals of the RHMT program is to create sustainable health student placement opportunities in rural and remote areas of Australia. The premise is that if student health professionals have positive placement experiences in rural and remote areas, they will be more inclined to seek employment in these areas once they have graduated. Complementing this is the long‐held notion that a health professional's decision to work in rural settings is influenced by having a rural background and family connections.^4^

Another approach to developing a rural and remote workforce is to enable students to complete their entire training in their rural or remote context—end‐to‐end training. This requires the provision of the educational infrastructure and personnel, including academics to be in the location the student is learning for the duration of the individual's learning. There is some evidence that this approach results in more professionals working in rural areas. In a comparison of dental graduate outcomes, Johnson et al^5^ reported 54% of graduates from a regional end‐to‐end program were working rurally, whereas 33% who did their training in a metropolitan program, with an extended rural placement, were working rurally after graduation. Thus, whilst several studies have been published on the location of graduates who have completed extended rural placements for medicine and some allied health disciplines, other than in dentistry, we are not aware of any data on the outcomes of end‐to‐end regional/ rural training of other allied health and nursing professions.

Therefore, the aim of this study was to describe the rural workforce outcomes for nursing, allied health and dental students who undertook end‐to‐end training in regional and rural Victoria noting the potential inclusion of a metropolitan‐based placement for speciality practice not available in rural or regional Victoria.

## 2 METHOD

### 2.1 Sample, data collection and analysis

The La Trobe University Human Research Ethics Committee confirmed this evaluation did not require research ethics approval. Therefore, we extracted a list of students in health disciplines that required registration with the Australian Health Practitioners Regulation Agency (AHPRA) to practice who graduated in dentistry, midwifery, nursing, oral health, occupational therapy, paramedicine, physiotherapy and podiatry from La Trobe Rural Health School, as well as pharmacy graduates (solely taught on a regional campus) and those graduating from the regional psychology program. All students who graduated between 2015 and 2019 were included. Using internal administrative databases, we extracted the number, duration and location of all placements undertaken by the students. Manual electronic searching of the publicly available AHPRA register of practitioners was then undertaken to identify each graduate's current recorded place of work. This address was then added to the other variables (address of origin, discipline, year of study completion, number, place and duration of placements) in the database. The school attempts to secure all placements in regional and rural areas. However, some students complete placements in metropolitan Victorian locations due to the low availability of some specialist placement experiences in regional Victoria.

### 2.2 Spatial methods

As postcodes do not map directly onto the Modified Monash Model (MMM), and to reduce human error in identifying the appropriate MMM code, we used automated geocoding to identity the rurality code for each location (student origin, placement location and registered primary place of practice).

The MMM classifications (an area‐level measure) were extracted for each location (an *x*,*y*‐coordinate) using a spatial join in ArcGIS. The MMM accounts for geographical remoteness using the Australian Statistical Geography Standard‐Remoteness Areas (ASGS‐RA), coupled with town size and road distances. The MMM was chosen for describing geographical access due to its contemporary policy relevance.^6^

### 2.3 Statistical analysis

Data on students' place of origin, placement location and place of work were coded into 4 categories, metropolitan, regional, rural or remote, based on the four categories in the MMM (metropolitan [MM1], regional [MM2], rural [MM3–MM5] and remote [MM6–MM7]). Using their placement data, we calculated the number of placements and the number of placement days the student completed in a regional, rural or remote area, and the number of placements and the number of placement days the student completed in a metropolitan area. As different programs have different requirements for placement days, we then calculated a ratio of rural to metropolitan days. To predict place of work after graduation, we ran a multiple regression analysis with predictors entered using a forward stepwise method (criterion for entry is the beta coefficient was significant at *P* < 0.05 level). Variables entered were years since graduation, rurality of origin, placement days in rural areas, number of placements in rural areas, number of placements in metro areas, placement days in metro areas and placement rurality index (ratio of rural to metro placement days).

## 3 RESULTS

Of the 5506 graduates from the La Trobe Rural Health School, 4153 were able to be matched on the AHPRA register. When stratified using the MMM, rural areas accounted for 40% of the graduates' place of origin, with almost 2/3 of midwives being from rural areas (Table 1). Overall, the primary place of practice (PPP) of graduates was split relatively evenly between metropolitan, regional and rural areas. Notably, 56% of graduate midwives, 38% of nurses and 35% of our paramedics were working rurally.

**TABLE 1:** Percentage of students from different locations at commencement of studies and primary place of work in 2020

	Number of students tracked	Origin				Primary place of practice (PPP)			
		Metropolitan	Regional	Rural	Remote	Metropolitan	Regional	Rural	Remote
Dentistry	327	70.0%	15.0%	14.7%	0.3%	70.2%	14.9%	14.9%	
Midwifery	164	6.1%	27.4%	65.9%	0.6%	13.4%	29.9%	56.1%	0.6%
Nursing	2224	11.4%	39.9%	48.4%	0.3%	23.3%	37.3%	38.3%	1.0%
Oral health	183	59.0%	19.1%	20.8%	1.1%	69.6%	16.0%	13.8%	0.6%
Occupational therapy	209	12.0%	44.5%	43.5%		23.6%	45.3%	30.7%	0.5%
Paramedicine	155	13.5%	45.8%	40.6%		31.2%	33.1%	35.7%	
Pharmacy	394	61.2%	17.0%	21.8%		66.0%	16.0%	17.6%	0.5%
Physio	314	38.5%	30.9%	30.6%		52.5%	27.8%	19.6%	
Podiatry	80	15.0%	40.0%	42.5%	2.5%	19.5%	41.5%	36.6%	2.4%
Psychology	46	23.9%	50.0%	26.1%		56.5%	30.4%	13.0%	
All students/graduates	4096	25.2%	34.2%	40.3%	0.3%	35.8%	31.7%	31.8%	0.7%

Note

Rural = Modified Monash Model 3–5; Remote = Modified Monash Model 6–7.

A summary of graduate primary place of practice in relation to student's origin shows that the vast majority of students are working in the same geographical classification they came from (Table 2). In addition, 16% of metropolitan‐origin students are now working in regional and rural areas, and 12% of regional‐origin students are working in rural areas. However, some metropolitan migration was evident, with 20%, 20% and 17% of regional‐, rural‐ and remote‐origin students, respectively, now working in metropolitan areas. During the 2015–2019 period, 45% of placements were in regional areas, and 48%, in rural Australia (Table 3). Rural placements were typically shorter than metropolitan placements, accounting for only 44% of placement days.

**TABLE 2:** Percentage of students working in different Modified Monash areas by origin

Home	Work			
	Metropolitan	Regional	Rural	Remote
Metropolitan	84%	8%	8%	<1%
Regional	20%	68%	12%	<1%
Rural	20%	15%	64%	<1%
Remote	17%	0%	25%	58%

Note

Rural = Modified Monash Model 3–5; Remote = Modified Monash Model 6–7.

**TABLE 3:** Number, number of days and percentage of location of student placements, 2015–2019

	Modified Monash Model	Number of placements	% of number of placements	Number of days	% of placement days
Metropolitan	1	585	7	9427	9
Regional	2	3760	45	48 590	46
Rural	3	1995	24	24 597	23
	4	1016	12	12 662	12
	5	992	14	9808	9
Remote	6	10	0.1	150	0.1
	7	8	0.1	115	0.1
Total		8366		105 349	

Multiple regression analysis indicates that where students are from is the strongest predictor of where they end up practicing, accounting for 33% of variance (see Table 4 for details of regression analysis). The number of years since graduation was a significant predictor of current work location, with the more years since graduation, the less likely they were to work rurally. The locations where students did their placement was also a significant predictor of their primary place of practice. The more days individuals spent on placement in a metropolitan area, the less likely they are to work in regional/rural areas, and the more placements they completed in rural areas, the more likely they are to work in regional and rural areas. That is on average if a student completes 22 weeks of placement in a metro location, they are likely to drop one level (MMM 2 to 1, or 3 to 2, or 4 to 3) in the rurality of their current workplace. However, years since graduation and placement location added an additional 2% of variance to the prediction of workplace location.

**TABLE 4:** Result of multiple regression analysis to predict workplace location category

	Unstandardised coefficients *B*		95.0% confidence interval for unstandardised coefficients *B*		Standardised coefficients	*t*	Sig.	Adjusted *R* ^2^
	*B*	SE	Lower bound	Upper bound	*β*			
All students								
Origin location	0.570	0.014	0.543	0.597	0.544	41.229	<0.001	0.33
Number of rural placements	0.047	0.007	0.033	0.060	0.103	6.625	<0.001	0.01
Number of placement days in a metro location	−0.006	0.001	−0.008	−0.003	−0.060	−4.567	<0.001	0.03
Number of years since graduation	−0.008	0.004	−0.016	0.000	−0.031	−2.012	.044	0.01
Allied health students								
Origin Location	0.542	0.019	0.505	0.578	0.553	28.774	<0.001	0.316
Number of years since graduation	−0.019	0.006	−0.031	−0.007	−0.063	−3.115	0.002	0.005
Number of rural placements	0.095	0.030	0.036	0.155	0.092	3.147	0.002	0.002
Ratio of rural to metro placements	−0.001	0.001	−0.003	0.000	−0.064	−2.203	0.028	0.002
Nursing students								
Home	0.558	0.022	0.516	0.601	0.482	25.636	<0.001	0.273
Number of placements days in rural location	0.003	0.001	0.001	0.005	0.103	3.482	<0.001	0.010
Number of placement days in a metro location	−0.018	0.004	−0.026	−0.010	−0.192	−4.200	<.001	0.008
Number of years since graduation	−0.021	0.007	−0.034	−0.008	−0.091	−3.174	0.002	0.002
Number of placements in metro location	0.164	0.066	0.034	0.295	0.114	2.471	0.014	0.002
Ratio of rural to metro placements	−0.001	0.000	−0.002	0.000	−0.073	−2.396	0.017	0.002

As nurses made up over 50% of the sample, we repeated the analysis separately for nursing and all other disciplines. The results for the two groups were ostensibly the same, and the same as for the whole sample. For nurses, two additional variables were significant predictors of work location, both relating to the measure of placement location, with 29% of variance in workplace location accounted for. However, the impact of metro placements is higher for nurses. On average, if a nursing student completes 8 weeks of placement in a metro location, they are likely to drop one level (MMM 2 to 1, or 3 to 2, or 4 to 3) in the rurality of their current workplace. For the non‐nursing students, whilst the actual time spent in metropolitan placements was no longer predictive of workplace location, the ratio of rural to metro placements was. This regression model accounted for 32% variance in workplace location.

## 4 DISCUSSION

Through extracting Modified Monash Model classifications based upon existing university records, and linking to AHPRA PPP data, we were able to identify the origin, placement location and place of work of over 4000 graduates of end‐to‐end rural programs for a diverse range of health disciplines. This approach addresses the issues of scale and policy relevance identified by Walsh et al.^7^, ^8^ Since its inception, La Trobe Rural Health School has trained more than 4000 health professionals, two thirds of which are now working in regional, rural and remote Australia, and as such is a major provider of the rural health workforce. By applying the MMM, these findings have direct policy relevance to the RHMT policy that is focused on areas outside MM1 (metropolitan) areas.

Whilst La Trobe Rural Health School regional campuses are largely based in regional cities, students undertook just over half of their placements in rural health services. Furthermore, although the strongest determinant of where graduates are now working is where they came from, it is important to note that where students did their placements also influenced where they work. These results show higher retention in rural workplaces than was reported for dentists completing end‐to‐end training previously,^5^ and much higher than is seen for the extended rural placement model most commonly advocated.^5^, ^9^

This speaks to the need to support regional and rural higher education capacity, as essential for training a sustainable rural health workforce. Victoria has four university departments of rural health; along the borders with New South Wales, there are two more university departments of rural health, and another on the South Australia/ Victoria border, resulting in a competition of securing rural placements for students. Therefore, we suggest that where there is conflict in providing placements, priority should be given for students undertaking end‐to‐end programs over students undertaking extended rural placements from metropolitan‐based programs.

**FIGURE 1:**
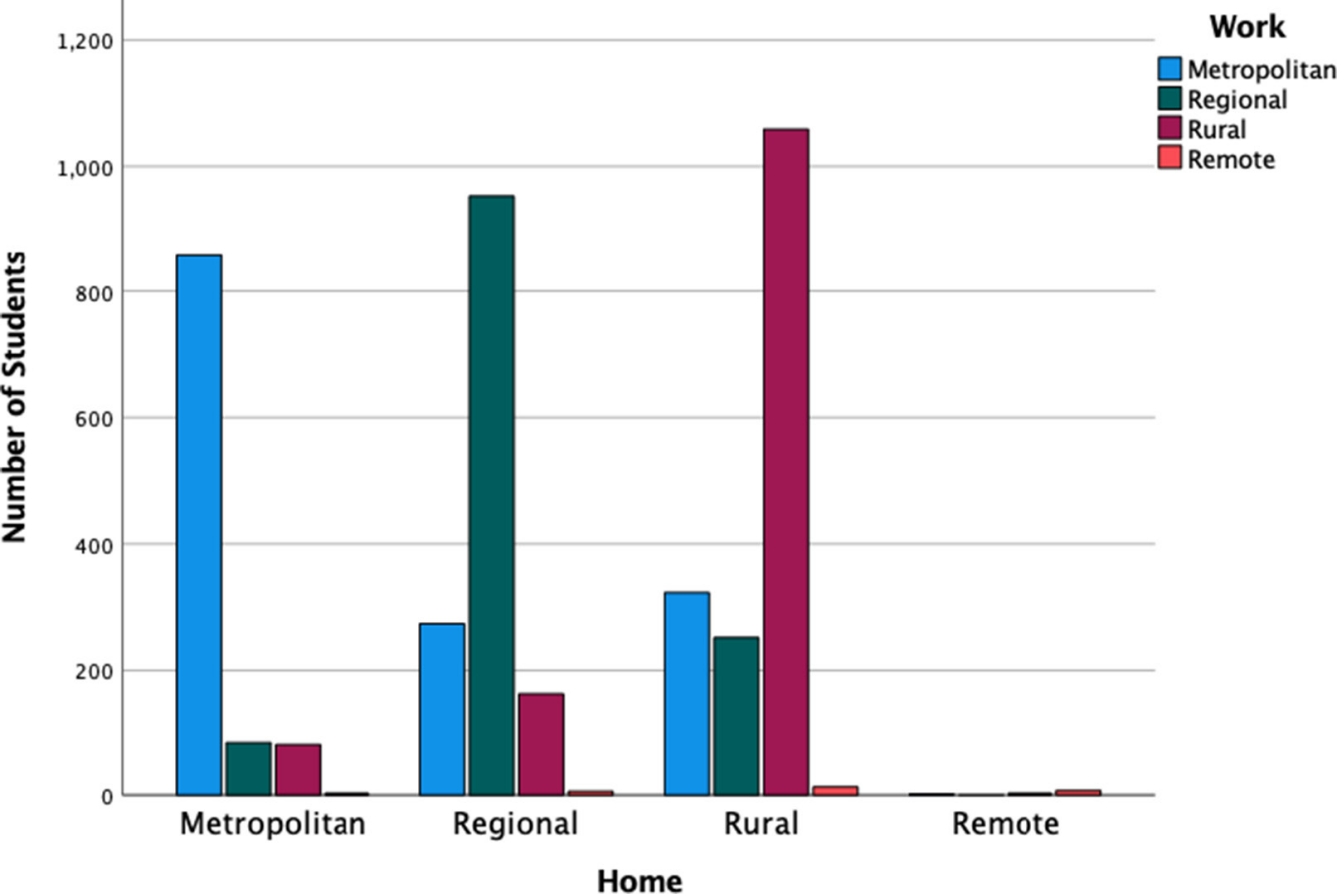
Students' current workplace location by location at the commencement of studies

However, it is also important to note that rural origin and rural study does not eliminate migration. About 20% of graduates in this study moved to work in metropolitan health service contexts (Figure 1), whilst there was only a 16% migration from metropolitan‐origin students. Thus, there is still a net loss to metropolitan areas. It is acknowledged that recent internal migration patterns counter the prevailing trend of migration of rural people to metropolitan areas^10^; however, more qualitative enquiry to understand this migration, what influences it and what options we can provide to minimise this is required. It could be these are temporary shifts to gain expertise or specialist knowledge not available in regional and rural areas—a longitudinal investigation is needed to better understand these dynamics. We also only located 75% of our graduates, with the remaining graduates unable to be located in the AHPRA register. This might be due to graduates changing careers, changing names and taking career breaks, but this is a loss to the system, and work needs to be done to understand these factors. This points to the need for longitudinal tracking of graduates from rural and metropolitan programs, such as work being undertaken in the NAHGOT study.^7^

## CONFLICT OF INTEREST

TCS, IKS, LS and MB are all employees of the La Trobe University.

## AUTHOR CONTRIBUTIONS

TCS: conceptualisation; formal analysis; writing—original draft. MB: writing—review and editing. VLV: formal analysis; methodology; writing—review and editing. LS: data curation; methodology; project administration; supervision. IS: conceptualisation; methodology; writing—original draft; writing—review and editing.

## References

[1] Australian Government DoH Modified Monash Model (MMM) 2019. In: editor. Available from https://data.gov.au/data/dataset/modified‐monash‐model‐mmm‐2019 Rural Distribution Section data.governance@health.gov.au; 2019. Accessed June 2021.

[2] Versace VL , Skinner TC , Bourke L , Harvey P , Barnett T . An analysis of the access measure adopted by national rural workforce policy: accounting for population distribution and socio‐economic status. Aust J Rural Health. 2021;29(5):801–810. 34672057 10.1111/ajr.12805

[3] Bourne K , Houghton K , How G , Achurch H , Beaton R . The big movers: Understanding Population Mobility in Regional Australia; 2020.

[4] Hegney D , McCarthy A , Rogers‐Clark C , Gorman D . Why nurses are attracted to rural and remote practice. Aust J Rural Health. 2002;10(3):178–86. 12081512 10.1046/j.1440-1584.2002.00447.x

[5] Johnson G , Wright FAC , Foster K . A longitudinal evaluation of the Rural Clinical Placement Program at the University of Sydney Dental School. Eur J Dent Educ. 2019;23(1):e59–e70. 30358039 10.1111/eje.12401

[6] Versace VL , Beks H , Charles J . Towards consistent geographical reporting of Australian health research. Med J Aust. 2021;215(11):525. 10.5694/mja2.51344 34773925

[7] Sutton KP , Beauchamp A , Smith T , Waller S , Brown L , Fisher K , et al. Rationale and protocol for the Nursing and Allied Health Graduate Outcomes Tracking (NAHGOT) study: a large‐scale longitudinal investigation of graduate practice destinations. Rural Remote Health. 2021;21(3):6407. 34587455 10.22605/RRH6407

[8] Walsh S , Lyle DM , Thompson SC , Versace VL , Browne LJ , Knight S , et al. The role of national policies to address rural allied health, nursing and dentistry workforce maldistribution. Med J Aust. 2020;213:S18–22.

[9] Brown L , Smith T , Wakely L , Little A , Wolfgang R , Burrows J . Preparing graduates to meet the allied health workforce needs in rural Australia: short‐term outcomes from a longitudinal study. Educ Sci. 2017;7(2):64.

[10] Jones M , Versace V , Lyle D , Walsh S . Return of the unexpected: rural workforce recruitment and retention in the era of COVID‐19. Aust J Rural Health. 2021;29:612–6. 34672056 10.1111/ajr.12817 PMC8652960

